# How to Get the Biggest Slice of the Cake. A Comparative View of Social Behaviour and Resource Access in Human Children and Nonhuman Primates

**DOI:** 10.3389/fpsyg.2020.584815

**Published:** 2020-11-03

**Authors:** Marjolijn M. Vermande, Elisabeth H. M. Sterck

**Affiliations:** ^1^Department of Child and Adolescent Studies, Utrecht University, Utrecht, Netherlands; ^2^Department of Biology, Utrecht University, Utrecht, Netherlands; ^3^Department of Animal Science, Biomedical Research Centre (BPRC), Rijswijk, Netherlands

**Keywords:** social hierarchy, social behaviour, alternative strategies, resource access, resource control, dominance, bonds, leadership

## Abstract

Social complexity results from engaging in different classes of social behaviour. The presence of different classes of social behaviour is reflected in multidimensional concepts of social asymmetry, found in both human and nonhuman primates. Based on an overview of such concepts, we propose that three classes of social behaviour are involved in having access to scarce and desired resources: next to aggressive and affiliative behaviour, also action indicating behaviour (i.e., inspire another individual to follow one’s example or intentions) may lead to resource access. Studies with nonhuman primate and human children show that the contribution of aggression and affiliation to resource access has been widely documented and that there is initial support for action indicating behaviour. In addition, the studies show similarities and differences in conceptualization and approach that may inspire future research. Future research should address the (in)dependency of the behavioural dimensions, their relative importance, individual differences in combined expression and the type of resources accessed. Only a multi-dimensional view on behaviour leading to resource access will highlight the benefits of social complexity.

## Introduction

Primates, both human and nonhuman, are characterized by complex social behaviours encompassing different classes of social behaviour. We explore how social complexity may be beneficial in accessing resources. In both human and nonhuman primates, unequal resource access is a pervasive feature of groups (Sapolsky, 2005). Individuals strive toward survival and reproduction in an environment that typically contains limited resources, which causes within-group competition (van Schaik, 1989; Pellegrini, 2008). Resources enable optimal reproduction and thus, biological fitness. However, in literature on human children, resources are not always directly linked to fitness, but often refer to coveted goods, partners, or features. Different types of resources can be distinguished, including *material* (e.g., food, treats, shelter, territory, toys, and money) and *social* (e.g., alliance, knowledge, tolerance, and affection) resources (Charlesworth, 1996; Keltner et al., 2003; Hawley, 2007; Pellegrini et al., 2011). Resources of one form (e.g., money, alliances) can be used as means of obtaining resources of another form (Charlesworth, 1996). Group members usually differ in their ability and motivation to prevail in resource competition (Charlesworth, 1996) and individuals may access resources by using different social strategies, based on different classes of social behaviour (Hawley, 1999; Overduin-de Vries et al., 2020).

Traditionally, aggression and the ensuing dominance relationships are considered to provide preferential access to resources (Sussman et al., 2005). This implicitly assumes that behaviour used for resource access is found in one behaviour dimension. More recently, however, it has been acknowledged that affiliative behaviours and eventual “bonds” [also called “good relationships,” and (among non-kin) “friendships”; Silk, 2002; Massen et al., 2010; Seyfarth and Cheney, 2012] may be effective as well in both humans and animals (Sussman et al., 2005). This leads to the proposal that social behaviour used for resource access is multidimensional. In particular in developmental psychology this view is commonly held and investigated (Santos and Vaughn, 2018). Moreover, we propose that a third class of social behaviour, namely inspiring the direction of action (indicating action) by esteemed individuals, may also lead to resource access.

The link between access to limited resources and social behaviours has been investigated in humans and animals, yet the concepts of social behaviour resulting in asymmetric resource access and findings of these two research fields are rarely compared directly. Although the study of resource access in developmental psychology originally has been influenced by sociobiology and ethology (e.g., Strayer and Strayer, 1976; Charlesworth, 1988; Hawley, 1999), there are some differences in approach. By identifying similarities, differences, and research gaps, we aim to stimulate research into different ways to access resources in humans and animals. More specific, we focus on comparing research on human children and nonhuman primates (further called primates) for three reasons: first, human and primate social behaviours may be similar when they are conserved and can be traced back to common ancestral behaviours; second, much detail of and variation in both primate and human children’s social behaviours is known, allowing a comparison; and third, the relationship between social behaviours and resource access has been topic of recent research on both human children and primates, providing theoretical and empirical frameworks that can be compared.

The central questions of the present conceptual analysis are: (1) whether three rather than two classes of social behaviour are conceptually linked to resource access for children and primates, (2) what empirical evidence relates classes of social behaviours to resource access in human children and primates, (3) what resources are accessed, and (4) how the different classes of social behaviours relate to each other.

## Conceptual Proposal: Three Classes of Social Behaviours can Lead to Resource Access

### Multidimensional Concepts of Social Asymmetry

There is no commonly accepted taxonomy of social asymmetry of individuals within groups. Many different concepts are used, both within and across disciplines (Henrich and Gil-White, 2001; Watts, 2010; Table 1). Although social asymmetry is often discussed in terms of aggression (e.g., the traditional view of dominance in terms of power-submission relations in dyadic contest and group structure; Lewis, 2002), recent developments in both primatology and social sciences indicate that non-agonistic classes of social behaviour may also be effective in gaining a high position.

**Table 1:** Multidimensional conceptualisations of social asymmetry, stressing either aggressive and affiliative behaviour or aggressive and action-indicating behaviour.

Concepts	Classes of social behaviour		
	**Aggressive behaviour** (inflicting damage or unpleasantness upon another individual)	**Affiliative behaviour** (binds individuals in (sub)groups by benefitting another individual)	**Action indicating behaviour** (inciting someone to follow his/her intention or example, because of the promise of success)
**Dominance** in terms of preferential access to scarce resources (“resource control”; Charlesworth, 1988; Hawley, 1999; Pellegrini, 2008)^a^	**Aggression**; **coercive resource control strategies**	**Co-operation** and other forms of **affiliation**; **prosocial** (positively assertive) **resource control strategies**	
**(Peer perceived) popularity**: power, prestige, or visibility in the peer group (Cillessen, 2011)^a^	**Antisocial behaviour** (aggression, disruptiveness)	**Prosocial behaviour** (voluntary behaviour to benefit someone else)	
**Social network centrality**: the most important or prominent actors within the network (Farmer and Rodkin, 1996; Sueur et al., 2011)^a^^,^^b^	**Antisocial behaviour** (aggression, disruptiveness)	**Prosocial behavioural** (cooperative, leading)	
**Power**: an asymmetrical dyadic relationship when preferences conflict (Lewis, 2002)^a,b^	**Dominance** (force or force threat)	**Leverage** (based on inalienable resources)	
**Dominant personality**: a personality trait focused on influencing others (Kalma et al., 1993)^a^	**Aggressive dominant** individuals use aggression to persuade others		**Socially-oriented dominant** individuals rely on reasoning in order to influence group decisions
**Status**: a hierarchy of rewards and/or displays (Henrich and Gil-White, 2001; Chapais, 2015)^a^^,^^b^	**Power** (force or force threat)		**Prestige** (freely conferred deference, drawn from reputation, respect, and reverence)
**Social hierarchy**: influence over others (Fragale et al., 2011)^a^	**Power** (the extent to which an individual can control others’ outcomes by granting or withholding valued resources)		**Status** (the extent to which an individual is respected, admired and highly regarded by others)
**Leadership**: non-random differential influence on group behaviour of conspecifics (Smith et al., 2016)^a^^,^^b^	**Dominance** (coercion to control the behaviour of subordinates)		**Visibility, knowledge, or other factors affecting voluntary decisions** to follow or emulate (i.e., prestige)

a These conceptualisations have been applied to humans.

b These conceptualisations have been applied to nonhuman primates.

Several concepts can be interpreted as considering both *aggressive* and *affiliative* social behaviour. First, in developmental science, *dominance* has been recently defined as successful competition over resources in the presence of others (labelled *resource control*; Charlesworth, 1988; Hawley, 1999; Pellegrini, 2008; Stump et al., 2009). Resource control encompasses both aggressive and positive, socially acceptable behaviours [such as (promising) reciprocity and cooperation]. Note that this use of dominance to describe the outcome of behaviour contrasts with the one-dimensional use of dominance by biologists and early ethologically oriented developmental psychologists, who define dominance in terms of asymmetry in agonistic conflicts. Second (peer-perceived), *popularity* in child peer groups is characterized by power, prestige, or visibility (Cillessen and Marks, 2011) and correlates with resource control (Olthof et al., 2011; Vermande et al., 2018). Popularity results from the strategic use of antisocial (e.g., physical attacks, gossiping, and bullying) and prosocial (i.e., voluntary behaviour to benefit another individual; Eisenberg et al., 2010) behaviours (Cillessen, 2011). Third, the concept of *social network centrality* partially overlaps with both resource control and popularity in children (Hawley, 2007; Cillessen and Marks, 2011; Zarbatany et al., 2019). Actors who are the most important or the most prominent are usually located in strategic locations within the network (Wasserman and Faust, 1994). Social network centrality as an index of social asymmetry is usually measured using affiliation networks. It is associated with both prosocial (e.g., cooperation, leading, and joking) and antisocial (e.g., picking on, teasing, and disruptiveness) behaviours in both children (Farmer and Rodkin, 1996; Gest et al., 2001) and primates (Lehmann and Ross, 2011; Sueur et al., 2011). Fourth, *power* refers to the ability to direct or influence the behaviour of others (Keltner et al., 2003; Giordano et al., 2006). Although it is often associated with aggression and dominance, some scholars argue that aggression is not the only component of power in humans and animals (Lewis, 2002, 2018; Smith et al., 2016). In this approach, power has two components: dominance (i.e., power based upon force or the threat of force) and leverage (i.e., power based on resources that cannot be taken by force, such as fertilizable eggs, services, and coalitionary support; Lewis, 2002, 2018). When an individual has leverage over an inalienable resource, others will have to use alternative methods to dominance to obtain the resource. We propose that to get access to individuals with leverage (i.e., a social resource), affiliative behaviour will be enlisted. In this view, power concerns aggressive and affiliative social behaviour.

Several other concepts of social asymmetry are considering both *aggressive* and *indicating action* behaviour (i.e., inspire another individual to follow one’s example or goals) rather than aggressive and affiliative behaviour. First, in humans, including young children, sometimes two subtypes of *dominant personality* – aggressive and social – have been identified. Both types demonstrate a focus on influencing others, but aggressive-dominant individuals use aggression (i.e., a more self-centred dictatorial type of strategy), whereas socially-oriented dominant individuals tend to make allies and try to sway others onto their side with solid arguments (Kalma et al., 1993; Cook et al., 2014). Second, *status* can be based on dominance (i.e., force or force threat) and on prestige (i.e., freely conferred deference; Henrich and Gil-White, 2001; Chapais, 2015; Cheng, 2020). Individuals with prestige achieve status by excelling in competences in valued domains (e.g., foraging success, physics, and basketball). Third, *social hierarchy* encompasses two potential influences over others, namely power, defined as “the extent to which an individual can control others’ outcomes by granting or withholding valued resources,” and status, defined as “the extent to which an individual is respected, admired and highly regarded by others” (Fragale et al., 2011, p. 767). Fourth, the related concept of *leadership* refers to non-random differential influence on group behaviour of conspecifics (Smith et al., 2016). Similar to power, differential influence may depend on dominance (i.e., coercion to control the behaviour of subordinates). Alternatively, it may depend on voluntary decisions to follow or emulate, for example, because of a leader’s visibility or knowledge. In such cases, a leader has prestige (Smith et al., 2016).

In conclusion, the overview makes clear that scholars have not converged on a common taxonomy of social asymmetry and that the same concepts may have different meanings. More important for our analysis is that the overview suggests that three rather than two classes of social behaviour may underlie social asymmetry.

### Aggressive, Affiliative, and Action Indicating Behaviours May Provide Access to Resources

**Figure 1:**
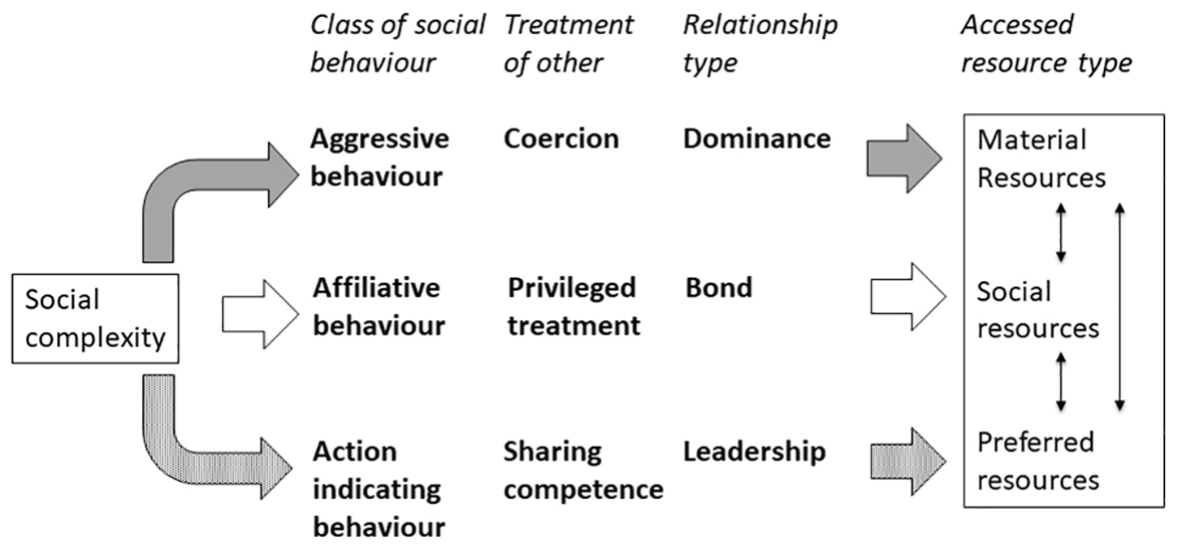
Proposed relations between three classes of social behaviour, the effect of this social behaviour on the interaction partner, the ensuing relationship when the pattern is consistent, and the type of resources that may be accessed. Social complexity results from expressing different classes of social behaviour.

As argued above, there are indications that three classes of social behaviour can result in social asymmetry and unequal resource access. These classes differ in how the receiving individual is affected and what type of social relationship (sensu Hinde, 1976, 1992) may result when these patterns are consistent over time (Figure 1). They may also provide access to specific types of resources. First, aggressive behaviour concerns the use of coercion to direct the behaviour of the receiving individual. The aggressive individual obtains preferential access to resources at the expense of the other individual. Consistent patterns in aggressive behaviour give rise to dominance relationships (as defined by biologists) at the dyadic level. Aggression will give access to resources (e.g., material resources like food and territories; or mating partners) by excluding competitors. Second, affiliative behaviour concerns giving the other individual a privileged treatment and therefore is beneficial to the receiver. The affiliative individual in turn can access resources that the receiver has to offer, such as sources of leverage (see section Multidimensional Concepts of Social Asymmetry). Both interaction partners may thus benefit and consistent patterns in affiliation lead to bonds, a proximate mechanism to achieve cooperation (de Waal and Luttrell, 1988) among kin (kin selection: Hamilton, 1964) and exchange of benefits among non-kin (reciprocal altruism: Trivers, 1971). The individual receiving affiliation can provide the affiliative individual with access to resources (e.g., social resources, such as support and tolerance, or material resources, such as toys). Third, an individual that exhibits action indicating behaviour can incite another individual to follow its example or intentions because of the promise of success. In this way, action indicating individuals can direct the behaviour of others to access preferred resources (e.g., doing a favourite game or visiting a favourite food tree, a common goal). Consistent patterns in indicating action identify leaders and followers. Thus, the three classes of social behaviour affect the receiving individuals in different ways and may lead to different types of social relationships (Figure 1). In addition, they all may provide access to resources, yet the type of resource may vary.

In the next section, we will explore whether empirical data support the conceptual idea that these three classes of social behaviour, that may be conserved in and shared by human children and primates, can contribute to resource access. If the three classes of social behaviour provide access to different resources, this indicates benefits of social complexity in both humans and primates.

## Three Classes of Social Behaviour and Resource Access: Empirical Links

In order to address the second research question, empirical studies on the relation between resource access and the three classes of social behaviour (aggressive, affiliative, and action indicating) are explored. While primatologists use observations of these behaviours, developmental psychologists use observations (often with pre-schoolers), but also peer reports (asking children as informants about their peers’ behaviour and/or resource access), teacher reports (using teachers as informants), and self-reports (asking children about their own behaviour and/or resource access). Resource access may be measured at the level of specific resource utilization (e.g., seconds spent viewing a cartoon in a competitive movie viewer procedure, Charlesworth, 1996; access to a new toy, Hawley and Bowers, 2018; contact with the other sex at monthly school dances, Pellegrini, 2008). Quite often, however, resource control is assessed using rating scales with items covering several aspects of resource control (e.g., getting first hold of the nicest toys or the best gadgets, being the centre of attention in a group, and getting what she/he wants), resulting in a measure of a child’s general resource access (e.g., Hawley, 2003; Olthof et al., 2011; Roseth et al., 2011; Ostrov and Guzzo, 2015). We will describe the types of resources accessed (research question 3) and the proposed relationships between the different classes of social behaviour leading to resource access (research question 4).

### Aggressive Behaviour

Aggression is commonly defined as behaviour inflicting damage or other unpleasantness upon another individual. In the human literature, it is often added that the perpetrator must believe that the behaviour will harm the target (in order to distinguish aggression from accidental harm or the by-product of helpful actions such as pain due to a dental procedure), and that the target is motivated to avoid the behaviour (to distinguish aggression from e.g., sexual masochism; Anderson and Bushman, 2002). Aggression can be used to displace another individual from a location or coerce an individual into an action. Therefore, aggression may enhance resource access. There is ample support for the link between aggressive behaviour and resource access.

#### Primates

Both aggressive interactions and dominance relationships are used to access resources. Dominance relationships are discerned when aggressive interactions of a dyad lead to a predictable winner and loser. Preferential access need not necessarily require aggression, since subordinates defer to dominants or avoid them (Schaub, 1995). There is broad evidence indicating that aggression and dominance can give access to food (Ostner and Schülke, 2014; Overduin-de Vries et al., 2020) and to mating partners (Alberts, 2012). One study reports that aggression can also give access to support in between-group conflicts (Arseneau-Robar et al., 2016). The effects of dominance relationships are also visible in fitness outcomes that result from resource access, where dominant males often have more offspring in a particular period (Alberts, 2012) and dominant females have a higher lifetime reproductive output (van Noordwijk and van Schaik, 1999; Pusey, 2012). Thus, aggression leads to individual fitness (Darwin, 1859) and aggressive behaviour is considered a straightforward method to access resources and is often implied when investigating resource access.

Dominance can also be used to coerce individuals. Coercion in a sexual context has been described for chimpanzees (*Pan troglodytes*: Smuts and Smuts, 1993; Muller et al., 2011; Feldblum et al., 2014) and orang-utans (*Pongo pygmaeus*: Knott et al., 2010), where males can force a female to mate. However, systematic coercion of group members is in general not effective (Clutton-Brock, 2002). Accordingly, forcing cooperation or grooming it is typically not successful (Overduin-de Vries et al., 2020), since the partner can withhold support or grooming. This leverage of cooperation partners (Lewis, 2002, 2018) or of individuals with specific competences (Chapais, 2015) requires other social strategies than aggression and dominance (Watts, 2010).

#### Human Children

Although aggression is often viewed by peer relation researchers as pathological, recent developments acknowledge that aggression can be functional for resource access (Pellegrini, 2008). An important difference in this regard is between reactive and proactive aggression. *Reactive aggression* is a reaction to a (presumed) threat that is associated with anger and emotion dysregulation. The function of this kind of behaviour is to defend oneself, not to access resources at the expense of others. *Proactive* or *instrumental* aggression is planned, goal-oriented, and unemotional. The function of this type of aggression is to take possession of things or to dominate or intimidate (Polman et al., 2009) and is positively associated with high rank (Stolz et al., 2016; van den Berg et al., 2019). For example, adolescents use aggression against same-sex peers (i.e., competitors) in order to obtain contacts with the other sex (Pellegrini, 2008).

A theory on the strategic use of behaviour is Resource Control Theory (RCT) of Hawley (1999). In this theory, *coercive resource control strategies* refer to proactive (instrumental) aggression (e.g., taking, demanding, threatening, and commanding). They appear early in life: toddlers and pre-schoolers predominantly apply aggressive strategies to obtain resources. Several studies have shown that applying aggression in terms of RCT’s coercive strategies is effective for obtaining above average resource access, both in preschool, childhood, and adolescence (Hawley, 2007, 2014, 2015; Reijntjes et al., 2018; Vermande et al., 2018). This also holds for bullying, a specific subtype of proactive aggression that is increasingly conceptualized as strategic behaviour motivated by a desire to gain a high position and resource access in the peer group (Olthof et al., 2011; Reijntjes et al., 2013a; Lee, 2020). Bully-victim relations resemble dominance-relations in that there is a power difference between perpetrator and victim.

#### Conclusion and Comparison

In primatology, aggressive conflicts and dominance relations are ways to exclude others from scarce resources. Developmental scientists stress that not all highly aggressive children have resource access. The type of aggression matters: resource access is positively associated with instrumental (proactive) rather than reactive aggression. In primatology, the accessed resources are typically identified, such as food and mating partners and one study mentions a support, while in developmental psychology what constitutes these resources is often more implicit or general. However, aggression may often not be effective in accessing *social* resources (see section Multidimensional Concepts of Social Asymmetry).

### Affiliative Behaviour

Affiliative behaviour binds individuals in groups (see section Aggressive, Affiliative, and Action Indicating Behaviours may Provide Access to Resources) and is related to social proximity and cohesion (Silk, 2002; Pellegrini, 2008). Like aggression, affiliative behaviour has been linked to resource access.

#### Primates

In primates, affiliation encompasses behaviours such as proximity, play, food-sharing, and huddling (Sussman et al., 2005; Wooddell et al., 2019), yet grooming is the most prevalent (Sussman et al., 2005) and will be the focus behaviour here.

The value of grooming has long been acknowledged, since it may be directed at valuable partners that can provide agonistic support (i.e., to form a coalition) during a conflict (Kummer, 1978). Note that in this scenario primates exchange one resource (support) for another (winning a conflict, obtaining a high rank or accessing a mating partner). In species with a despotic dominance hierarchy grooming is preferentially directed at higher-ranking individuals to secure their coalitionary support (Seyfarth, 1977; Schino, 2001), while grooming is not related to rank differences in species with an egalitarian dominance hierarchy (Leinfelder et al., 2001). This indicates that bonds can affect dominance relationships and that these may be correlated. However, aggressive and affiliative behaviour can independently lead to resource access (Overduin-de Vries et al., 2020). Similarly, personality research indicates that the expression of aggressive and affiliative behaviour load on different axes and may be independent (Koski, 2011; Seyfarth et al., 2012; Wooddell et al., 2017; Ebenau et al., 2020).

Grooming can be used to obtain direct access to a resource. The value of grooming as a commodity to obtain direct benefits has been theoretically deduced in Biological Market Theory (BMT; Noë et al., 1991; Noë and Hammerstein, 1994). The distribution of grooming among group members will depend on the value of the resource that others have to offer and on the number of other individuals that offer this resource. An individual that has leveraged since it can provide a rare commodity (e.g., the only individual that can open a box with food; Fruteau et al., 2009) has a higher value than when more individuals can provide access. Therefore, the provider of the rare commodity can demand a higher payment for this commodity, for example, longer grooming. Accordingly, an individual with leverage over a relatively rare resource (e.g., grooming: Schino and Aureli, 2009, food: Fruteau et al., 2009, and infants: Henzi and Barrett, 2002) can obtain more grooming. BMT is often considered to explain short-term exchanges of grooming and resource access (Henzi and Barrett, 2007).

Systematic grooming of particular individuals will lead to bonds (Silk, 2002; Massen et al., 2010; Seyfarth and Cheney, 2012; Evers et al., 2014, 2015). Although the existence of bonds has long been acknowledged (e.g., Kummer, 1978; Wrangham, 1980), only the last 15 years evidence emerged that bonds provide fitness benefits (Nishida and Hosaka, 1996; Palombit et al., 1997; Silk et al., 2006a,b; Kulik et al., 2012; Massen et al., 2012). The proximate mechanisms responsible for these benefits are often not addressed (Thompson, 2019). For females these mechanisms may concern the proximity patterns and ensuing safety, for males enhanced interest from mating partners.

Affiliative behaviour may also repair the harmful effects of aggression. de Waal and van Roosmalen (1979) were the first to notice that chimpanzees do not necessarily keep distance after a conflict, but that the former opponents can affiliate with each other. They proposed that this constituted reconciliation. Reconciliation has been found in numerous other primates and animals (Aureli and de Waal, 2000) and functions to reduce stress, prevent renewed aggression, and repair the relationship (Aureli et al., 2002). Reconciliation is especially found among family members and among individuals with bonds (Aureli et al., 2002).

Effects of bonds have been found, while controlling for dominance (e.g., Silk et al., 2006a, 2010), indicating that affiliative strategies cannot be explained solely on the basis of dominance and that these two social strategies have separate effects. However, dominance may overshadow bonds, since in particular settings dominance may be more important (Fruteau et al., 2009; Massen et al., 2011), suggesting that a bond concerns the voluntary bestowing of benefits. The settings where bonds are or are not expressed have not been addressed systematically, but dominance may overrule good relationships in particular in situations of high contest competition. This suggests that contest for a resource should not be high, but possibly intermediate or low, for bonds to have effect. In addition, there is ample evidence that bonds lead to cooperative coalitions (Schino, 2007; Schino et al., 2007), suggesting that bonds provide benefits in contest competition for monopolisable but shareable resources. Altogether, this suggests that aggressive and affiliative strategies may be correlated, but also may operate independently (Wooddell et al., 2017).

#### Human Children

In developmental psychology, aggression and affiliative behaviour have traditionally been placed at opposite ends of a continuum (Hawley et al., 2007a; Pellegrini, 2008). More recently, however, it has been acknowledged that aggression and affiliation may share the same function (i.e., gaining resources; Table 1).

In line with primatologists, traditionally most scholars defined dominance in terms of aggression and power-submission relations. However, in the 1970s and 1980s some human ethologists – inspired by personal observations, evolutionary theory (e.g., Trivers, 1971), and social exchange theories – suggested that cooperative behaviours may also contribute to establishing dominance (e.g., Crook, 1971; Strayer and Strayer, 1976; Charlesworth, 1988).^1^ For example, it was observed that a relatively low-aggressive preschool girl was the most high-ranking individual in the group, suggesting that cooperative interactions play a role in young children’s hierarchical relations (Strayer and Strayer, 1976, p. 987–988). This possibility was investigated with respect to level of resource utilization in young children (i.e., watching a movie through a movie viewer). It was shown that *cooperation* can function as competition over limited resources (Charlesworth, 1982, 1988, 1996), next to being aggressive. Young children who combined cooperation with coercion were most effective in achieving viewing time (e.g., Charlesworth and Dzur, 1987; Charlesworth, 1996; Green et al., 2003; Table 1).

Some scholars posit that at the beginning of a new group, individuals predominantly resort to aggression to *establish* resource control, but over time engage in lower levels of aggression and increase their use of prosocial behaviour to *maintain* it (Pellegrini, 2008; Pellegrini et al., 2011). Partial support has been found for this hypothesis. For example, in a study across the school year, pre-schoolers’ aggressive behaviour decreased over time, but positive interactions remained relatively high in pre-schoolers scoring high on resource control. A noticeable example of positive interactions concerned reconciliation: pre-schoolers high on resource control were more likely to initiate reconciliation after also initiating an aggressive conflict: they appeared to reconcile strategically as a way to keeping defeated peers as affiliates (or simply to prevent that defeated peers would defame them to others; Roseth et al., 2011). In another study, other aspects of prosocial behaviour (e.g., sharing, helping) predicted an increase in teacher-rated resource control after 4 months in early childhood (Ostrov and Guzzo, 2015). Unfortunately, this study did not control for the effect of aggression. Contrary to Pellegrini’s hypothesis, resource control was not a predictor of changes (i.e., increase) in prosocial behaviour over time (Ostrov and Guzzo, 2015).

According to RCT, two broad classes of strategic behaviours to acquire resources can be distinguished (Table 1): *coercive* (section Aggressive Behaviour) and *prosocial resource control strategies*. Prosocial strategies appear around the age of five, as verbal abilities and social skills to negotiate with peers develop (Hawley, 1999). Prosocial strategies pertain to positive, socially acceptable behaviours used to obtain resource control, including promising reciprocity and cooperation (e.g., trading toys, promising friendship, and helping someone who does not need help). It should be noted that according to RCT, prosocial strategies are self-serving rather than other-serving or altruistic (Hawley, 2014, 2015). They are instrumental (Hawley and Bowers, 2018) and could be described as “positively assertive” (Vermande et al., 2018). Nevertheless, prosocial strategies are supposed to shape bonds with others. Using various methods and age groups (e.g., observations of dyads competing for a novel toy; rating scales with different informants), coercive and prosocial strategies vary from being mildly to strongly positively correlated. Negative correlations between the two types of strategies were never found, suggesting a common function (i.e., obtaining resources; Hawley, 2007; Hawley and Bowers, 2018). In addition, both coercive and prosocial resource control strategies are effective for obtaining above average resource control (Hawley, 2007; Hawley and Bowers, 2018). However, in spite of being correlated, others have found that the direct effect of prosocial strategies compared to coercive strategies is small in children and adolescents (Bassa, 2018; Vermande et al., 2018). Coercive and prosocial strategies may interact, explaining additional variance (Bassa, 2018).

RCT classifies participants into five different subtypes typically using *a priori* cut-off points (Hawley and Bowers, 2018). *Prosocial controllers* primarily use prosocial strategies, *coercive controllers* primarily use coercive strategies, *bistrategic controllers* score high on both, *noncontrollers* score low on both, and the remaining *typicals* score neither low nor high on both strategies. These subtypes show longitudinal stability from grade 4 to 6 (Reijntjes et al., 2018). Bistrategic controllers across all age groups are the most successful in acquiring resources, followed by prosocial and coercive controllers, whereas typicals and noncontrollers have only average and negligible/low levels of resource control, respectively (Hawley, 2014, 2015). However, some studies showed bistrategics to score as high on resource control as prosocial controllers (Hawley, 2003) or coercive controllers (Reijntjes et al., 2018), and prosocials and typicals do not always differ in resource control (Olthof et al., 2011; Reijntjes et al., 2018). In addition, bistrategics, like prosocial controllers, are sought out for friendships and are liked by peers (Hawley and Bowers, 2018), but others found that they evoked clear negative peer reactions (e.g., enmity nominations, low likeability; Reijntjes et al., 2018).

To what extent prosocial *behaviour* and instrumental prosocial resource control *strategies* overlap, is not clear and need further scrutiny (Ostrov and Guzzo, 2015). In addition, correlations between aggressive and affiliative behaviour may be lower if the items do not cover instrumentality, but only refer to the form of the behaviour.

#### Conclusion and Comparison

In both primate and human studies, affiliative behaviour can lead to resource access. While in primate studies affiliative relationships are determined independently of dominance and in human studies they are traditionally considered opposite ends of a continuum, both fields recently acknowledged that both may be correlated. Nevertheless, in primatology aggression and affiliation are usually studied in isolation, except the special case of studies concerning reconciliation, whereas in developmental psychology often both types of behaviours and their relative importance are examined. Results show that the combined use of aggression and affiliation by one individual may enhance resource access. A person-centred approach is unfamiliar in primatology. However, the two fields differ in identifying the accessed resources. In primatology, empirical outcomes indicate that affiliative behaviours can lead in several ways to resource access: they can access services, such as coalitionary support, and these services can affect relationships, such as enhance dominance, and services can lead to or be exchanged for resources, such as food or mating partners. These are often, but not necessarily, social resources. Although in developmental psychology some studies measured access to specific resources (e.g., a toy and a movie viewer), often a measure of a child’s general resource access effectiveness (resource control) is used.

### Action Indicating Behaviour: Inspiring Followers to Follow the Lead

Action indicating behaviour refers to inspiring followers to conduct particular behaviour that the leading individual wants. It is probably based on reputation, prestige, or peer regard (Vermande et al., 2018). Action indicating behaviour and its connection with resource access has received little explicit attention, but was recently explored in children (Vermande et al., 2018). In primates, the role of this behaviour has not been specifically addressed, but there are related studies on leadership and prestige (Chapais, 2015; Smith et al., 2016). Therefore, we start this section with human children and then discuss primates. There are some first indications that action indicating behaviour may lead to resource access.

#### Human Children

Action indicating behaviour involves inspiring others to follow one’s example (e.g., by social learning or modelling; Henrich and Gil-White, 2001) or intentions (e.g., a common goal, joint actions) because of the promise of success. This behaviour has been theoretically linked to leadership in organizations (Judge et al., 2009; Hogan and Blickle, 2018). As leadership is a measure of social asymmetry (see section Multidimensional Concepts of Social Asymmetry), action indicating behaviour may also be associated with resource access. That is, children who are able to indicate action for group members may be more proficient in getting what they want.

Vermande et al. (2018) examined the degree to which action indicating behaviour, referred to as “inspirational behaviour,” was associated with resource control above and beyond the effects of RCT’s (see sections Aggressive Behaviour and Affiliative Behaviour) coercive and prosocial strategies in young adolescents. They used peer ratings of strategy use and resource control. The action indicating behaviour items related to trying to get others enthusiastic about something, convincing others, and persuading others by giving suggestions. Confirmatory factor analyses indicated that the prosocial and action indicating strategy items, which refer both to socially accepted behaviour, loaded strongly on their own unique factor, and thus are different dimensions. All three classes of social behaviour were positively correlated with each other and with resource control, supporting the view that the three behaviours share a common underlying function. In addition, all three classes of behaviours were positive statistical predictors of resource control, but both coercive and action indicating behaviour were moderately strong predictors, whereas prosocial strategy use was a relatively weak predictor of resource control. Adolescents with prestige may be especially adept in using action indicating behaviour effectively (Vermande et al., 2018).

#### Primates

Several lines of research suggest that primates may show action indicating behaviour as well. Firstly, the related concept of prestige (excellence or competence in valued domains; see section Multidimensional Concepts of Social Asymmetry) has also been linked to dominance in primates and the social learning processes involved in prestige (i.e., followers copying the behaviour of expert role models) may also be relevant in primates (Chapais, 2015). In addition, the concept of travel leadership has been amply studied in primates (Smith et al., 2016) and may be related to action indicating behaviour. Moreover, the concept of “reputation,” that follows from the theory of indirect reciprocity, where a beneficial act to one individual results in a beneficial act to the original benefactor by a non-involved individual (Nowak and Sigmund, 1998), may apply. Thus, we will explore the link between action indicating behaviour, competences, social learning, travel order, and reputation.

Chapais (2015) reacted on the claim that only humans have status based on dominance and prestige (Henrich and Gil-White, 2001) by substantiating that also primates can exert these two routes to achieve status (see section Multidimensional Concepts of Social Asymmetry). However, in primates these prestige-related competences are derived from or correlated with coercive dominance. They have become independent only in humans, since human cumulative culture has led to many domains of non-competitive competences (e.g., making tools, dealing with the supra-natural, and dancing). Nevertheless, he argued that these two classes of social behaviour both lead to status and that also in primates action indicating behaviour is related to resource access. How these two types of behaviour relate to one another, whether they are indeed always closely linked or can be independent, has to be further explored. Moreover, the benefits of competence-based dominance remain to be established.

As action indicating behaviour or prestige incites others to follow example, studies on social learning may be informative. In these studies, a demonstrator shows a behaviour and followers may copy it. Demonstrators that are dominant (Horner et al., 2010; but see Botting et al., 2018), familiar (Perry, 2011), more knowledgeable (van de Waal et al., 2010) or obtain more benefits (Bono et al., 2018) are more readily copied. Knowledge of a demonstrator’s abilities results from the observation of their earlier performance, i.e., its reputation that may not affect the observer directly. Therefore, a demonstrator’s behaviour and its effect on the observer can highlight processes involved in indirect reciprocity. In addition, the characteristics of copied demonstrators may inform us on the competences that primates express (cf. Chapais, 2015) and that lead to following. While apes can observe the reputation of others (Herrmann et al., 2013), they may not manage their own reputation (Engelmann et al., 2016). One benefit for demonstrators has been found: being knowledgeable may lead to obtaining bonds independent of dominance (ring-tailed lemurs: *Lemur catta*; Kulahci et al., 2018).

Leadership in primate travel orders (Petit and Bon, 2010; King and Sueur, 2011) may be viewed as a specific example of indicating action to group members. Travel orders may be started by dominants or may be distributed over multiple group members (review: King and Sueur, 2011). However, coercive dominance will not be required in inciting group travel, since typically others will follow the “lead” of an individual without this leader forcing or enticing followers (Conradt and Roper, 2005). Therefore, leadership is potentially independent from aggressive and affiliative social behaviours. Indeed, followers follow not only dominants (Watts, 2000; Rowe et al., 2018), or kin (Leca et al., 2003; Rowe et al., 2018) and close affiliates (Wang et al., 2016), but also central group members (Leca et al., 2003). The ecological setting may also determine who is leading the travel order: when food sources are monopolisable dominants lead, while leadership is distributed with non-monopolizable food sources (King et al., 2008). The leader may obtain most benefits through enhanced access to food (King et al., 2008) or may not directly benefit from its behaviour (Smith et al., 2016). The benefits for followers may concern group cohesion. Altogether, whether travel leadership benefits the participants, either leader or follower, has received only limited attention. In addition, the individual competences of leaders (cf. Chapais, 2015) should be determined. In any case, also in primates action indicating behaviour in the form of travel order leadership may be different from aggression and affiliation.

#### Conclusion and Comparison

Action indicating behaviour appears to be a third way to access resources in young human adolescents, above and beyond aggressive and affiliative behaviour. Others may follow inspirational individuals due to their competences (prestige), yet what constitutes competences needs systematic attention. Primate literature on the topics of prestige, leadership, and social learning suggests that action indicating behaviour may gain specific resources, such as preferred food or obtaining bonds. In the study with human adolescents, resource access was measured as general resource control. Altogether, action indicating behaviour as a means to gain resource access is relatively new and has been little investigated.

## Discussion

This conceptual analysis highlighted that resource access can conceptually be linked to three different classes of social behaviour (research question 1; Figure 1) and explored empirical evidence that actually links these classes of social behaviour to resource access (research question 2), what resources are accessed (research question 3) and how these different classes of social behaviour are related (research question 4).

### Three Classes of Social Behaviours Are Empirically Linked to Resource Access

Empirical studies on the link between resource access and the three classes of social behaviour show that the contribution of aggression and affiliation to resource access has been widely documented (research question 2). However, in primatology, aggression and affiliation are usually studied in isolation – except the special case of studies concerning reconciliation – whereas in developmental psychology often both types of behaviours and their relative importance are examined. Results show that the combined use of aggression and affiliation may enhance resource access.

As yet only initial empirical support exists for the link between action indicating behaviour and resource access. Its contribution to resource control in human children has been found in one study showing that inspiring others is indeed a separate dimension, that it is correlated to both aggressive and affiliative behaviour, and that it explains a significant portion of variance in resource control above and beyond aggressive and affiliative behaviour (section Action Indicating Behaviour: Inspiring Followers to Follow the Lead). These results require further support. In addition, what makes a child inspiring to followers should be investigated. Prestige (i.e., competence in a valued domain) may be relevant. In primates, the relation between action indicating behaviour and resource access is implicit in several studies concerning prestige, social learning, and travel order and needs to be further explored. Research is also needed to understand the temporal relations between social behaviours and resource access, for example, to investigate how effective resource access leads to the use of more action indicating behaviour and receiving more affiliative offers from interaction partners over time.

### The Classes of Social Behaviours and Specific Types of Resources

The three classes of social behaviours may access different types of resources (research question 3). In developmental psychology, the nature of the accessed resources is typically less clear than in primatology. This difference is due to the different methods: while primatology has to employ observational methods, in developmental psychology often questionnaires are used that do not necessarily specify resources or combine them in an umbrella term like “resource control.” We cautiously suggested (Figure 1) that different classes of social behaviour may be employed to access different types of resources. Although primate literature indicates that specific behaviour may lead to specific resources (i.e., aggressive behaviour provides access to food, while affiliative behaviour provides access to social resources), also different connections are found. Both aggression and affiliation can provide access to mating partners, aggression results in excluding competitors for a mating partner and forcing of a mating partner, whereas affiliation may serve to overcome leverage from the mating partner. Also support, a social resource that is often obtained through grooming but can also be required through aggression, can lead to access to food, defending a territory and mating partners. Thus, access to resources can be a direct consequence of social behaviour, or one type of resource can be exchanged for another (indirect) and such exchanges of resources may even happen several times (grooming for support for dominance for access to food). In short, the link between a class of social behaviour and type of resource accessed appears to be variable and may be indirect. The connection between social behaviour and type of resource has received little attention in developmental psychology, although Pellegrini (2008) argues that in scramble situations, characterised by relatively abundant resources, individuals are less likely to use aggression than affiliation. However, systematic exploration of the connection between class of social behaviour and resource type, and whether indicting action leads to access of even different resources (e.g., joint actions that the leader wants or pursuits), has to be conducted. Both the typical connection between a class of social behaviour with a type of resource and the use of alternative social behaviours to access similar resources, indicate that the ability of an individual to show all these behavioural alternatives, i.e., social complexity, will provide benefits.

### Combination of the Classes of Social Behaviours

The three classes of social behaviours can be related in different ways: diametric opposites (negatively correlated), independent (not correlated), or co-expressed (positively correlated; research question 4). As described in section Three Classes of Social Behaviour and Resource Access: Empirical Links, several studies show that aggression and affiliation (and one study that aggression, affiliation and action indicating behaviour) positively correlate, whereas others indicate that they are independent. We found no studies suggesting that the behavioural dimensions are opposites.

With the exception of reconciliation, in primatology aggression and affiliation are usually studied in isolation or when controlling for dominance, whereas in developmental psychology often several types of behaviours and their relative importance are examined (section Three Classes of Social Behaviour and Resource Access: Empirical Links). This may be done using a *variable-centred* approach, including investigating children’s social behaviours as predictors of resource control. Another way to study the effects of social behaviours is to distinguish subtypes or groups of children, where an individual is characterised based on the relative use of one or more types of behaviours. This *person-centred* approach has been examined with aggressive and affiliative behaviour; combinations with action indicating behaviour are yet to be explored. Results typically show that children who combine aggression and affiliation (i.e., “bistrategic controllers”) are particularly effective resource controllers (see the RCT in section Affiliative Behaviour). However, whether this superiority in resource control is associated with other benefits such as psychosocial health is not clear. For example, bistrategic adolescents have been found to display lower than average levels of self-esteem and mental health over time (Ciarrochi et al., 2019) and noncontrolling children, although the lowest on resource control, do not always experience negative evaluations by peers (Reijntjes et al., 2018). A person-oriented approach is relatively new in primate research and may yield interesting insights.

Several scholars suggest *social competence* as a central concept in integrating the different classes of social behaviour (e.g., Strayer, 1989; Hawley, 2007; Vaughn and Santos, 2009). Some primate studies indicate that specific individuals may be able to use both aggressive and affiliative behaviour to access resources (e.g., Overduin-de Vries et al., 2020). In human children, effective resource access may be attributed to the flexible, strategic combination of social behaviours in different competitive contexts and/or with different interaction partners (Hawley, 1999; Pellegrini et al., 2011). Children high on resource control are assumed to have sophisticated social-cognitive skills that allow them to execute social behaviours successfully (Hawley et al., 2007a,b; Vermande et al., 2018). These skills may include abilities in the cognitive processing of emotions and the interpersonal regulation of emotion as recent studies suggest (Engel-Yeger et al., 2016; De Berardis et al., 2017). However, most studies rather focus on indicators of social competence (e.g., the combination of high status and good peer relations). Exactly what skills are involved has been rarely examined.

### Implications for Education

Several implications and issues emerge from the above discussion. First, school policy should not only emphasize academic performance, but also focus on socialisation opportunities that are necessary for the development of social competence (Liu et al., in press). These include offering non-academic classes (e.g., drama, music, and art; Stump et al., 2009), using collaborative learning techniques (Laal and Ghodsi, 2012), and encouraging pupils to experience leadership roles (Karagianni and Montgomery, 2018). Second, teachers have the difficult task of protecting the interests of both individual children and their peers. On the one hand, as aggression can be functional and may not be harmful to the social well-being of the child (section Affiliative Behaviour), the question is whether teachers should strive to root out all aggression in all children (Stump et al., 2009). On the other hand, bullying a specific subtype of aggression should not be tolerated. Because bullying is an effective way to gain resource access and popularity in the peer group (section Aggressive Behaviour) with little personal costs (Reijntjes et al., 2013b), bullies are likely to have little motivation to change their behaviour. Hence, intervention is most likely to be effective when bullying becomes less rewarding (e.g., fostering anti-bullying attitudes in the peer group, Polanin et al., 2012) and bullies are taught more acceptable ways to achieve resource access and popularity (Ellis et al., 2016). At the same time, offering children safe strategies to standing up for victims as well as teacher intervention in stopping bullying incidents and supporting the victim are important (Salmivalli et al., 2010; Swift et al., 2017). At a more general level, teachers should help children who are permanent losers of resource competition (e.g., by fostering social skills). Intervening by teachers starts with teachers being able to recognize the dynamics of competition for resources within classrooms.

## Conclusion

There are theoretical and empirical arguments for three classes of social behaviour being involved in having access to resources. Empirical support for action indicating behaviour is limited, however. Future studies should address the (in)dependency of the behavioural dimensions, their relative importance, individual differences in combined expression and their specific benefits. The ability to use all three classes of behaviour, and thus social complexity, appears to be highly beneficial. Altogether, social behaviour and its link to resource access seem to be alike in primates and human children, and differences between the research fields identified novel avenues of research.

## Author Contributions

Both authors listed have made a substantial, direct and intellectual contribution to the work, and approved it for publication. The authors share first authorship.

### Conflict of Interest

The authors declare that the research was conducted in the absence of any commercial or financial relationships that could be construed as a potential conflict of interest.

## Footnotes

^1^The strategic use of aggression and cooperation shares features with social exchange theories which posit that social behaviour is driven by subjective cost-benefit analysis and the comparison of alternatives (Green and Rechis, 2006). However, social exchange theories do not have aggression and cooperation for resources as a central theme (Charlesworth, 1996)
